# A Beginner’s Guide to Collecting Questing Hard Ticks (Acari: Ixodidae): A Standardized Tick Dragging Protocol

**DOI:** 10.1093/jisesa/ieaa073

**Published:** 2020-11-02

**Authors:** Jordan Salomon, Sarah A Hamer, Andrea Swei

**Affiliations:** 1 Department of Veterinary Integrative Biosciences, Texas A&M University, College Station, TX; 2 Department of Biology, San Francisco State University, San Francisco, CA

**Keywords:** tick, dragging, tick collection, protocol, medical entomology

## Abstract

Tick-borne diseases are emerging globally, necessitating increased research and coordination of tick surveillance practices. The most widely used technique for active collection of host-seeking, human-biting tick vectors is ‘tick dragging’, by which a cloth is dragged across the top of the vegetation or forest floor and regularly checked for the presence of ticks. Use of variable dragging protocols limits the ability of researchers to combine data sets for comparative analyses or determine patterns and trends across different spatial and temporal scales. Standardization of tick drag collection and reporting methodology will greatly benefit the field of tick-pathogen studies. Based on the recommendations of the Center for Disease Control and Prevention and other ecological considerations, we propose that tick dragging should be conducted to sample at least 750 m^2^ along linear transects when habitat allows in a manner that reduces bias in the sampled area, and report density of each tick species and life stage separately. A protocol for constructing a standard drag cloth, establishing linear transects, and drag sampling is presented, along with a downloadable datasheet that can be modified to suit the needs of different projects. Efforts to align tick surveillance according to these standard best practices will help generate robust data on tick population biology.

Tick surveillance in medical and veterinary entomology began in 1902 (Grutzner 1902), when *Ixodes ricinus* (Linnaeus) were collected from sheep to understand their effect on sheep health. While early efforts focused on collecting ticks directly from their bloodmeal hosts, methods of collecting ticks off-hosts were eventually developed to monitor tick populations during their host-seeking period, especially as their importance as vectors of agricultural and human pathogens grew (Milne 1943; Falco and Fish 1988, 1992; Daniels et al. 2000). Methods such as flagging, dragging, CO_2_ baiting, sweeping, and the use of human sentinels have all been employed for surveillance of host-seeking ticks, and specific sampling methodology is highly variable among research groups depending on the research question or surveillance objective (Falco and Fish 1992, Schulze et al. 1997, Gherman et al. 2012, Chong et al. 2013, Eisen and Paddock 2020, Kugeler and Eisen 2020).

Different methods for collecting host-seeking ticks are used because the life history of some tick species necessitate specialized collection techniques. For example, the lonestar tick, *Amblyomma americanum* (Linnaeus), a vector of *Rickettsia rickettsia* (Brumpt) (Rickettsiales: Rickettsiaceae), *Borrelia lonestari* (Swellengrebel) (Spirochaetales: Spirochaetaceae), and *Francisella tularensis*, (McCoy and Chapin) (Thiotrichales: Franisallaceae) is an aggressively mobile tick and CO_2_ traps can effectively lure host-seeking ticks from the local environment (Childs and Paddock 2003, Petry et al. 2010). Nidicolous tick species like *Ixodes angustus* (Neumann), which is closely associated with rodent hosts, commonly reside in the burrows and nests of their hosts (Easton and Goulding 1974, Furman and Loomis 1984), so their collection necessitates destructive sampling of the host environment or on-host collections (Foley et al. 2011). Other tick species are classified as ambush or ‘sit-and-wait’ predators that quest to the top of vegetation and wait to attach to a host as they pass by (Waladde and Rice 1982). This includes the blacklegged ticks, *Ixodes scapularis* (Say) and *Ixodes pacificus* (Cooley and Kohls), both vectors of the Lyme disease pathogen and several other emerging pathogens (Steere and Malawista 1979, Burgdorfer et al. 1985, Spielman et al. 1985, Lane and Burgdorfer 1987, Costero and Grayson 1996, Mun et al. 2006, Teglas and Foley 2006, Barbour et al. 2009). Accordingly, for these ‘sit-and-wait’ ticks, collection methods that provide a large surface area to contact and collect these ticks are useful.

Host-seeking within the Ixodidae family is facilitated by the tick’s Haller’s organs, a pair of sensory organs located on the first segment of their first pair of legs (Nuttall et al. 1908, Waladde and Rice 1982, Steullet and Guerin 1992). When host-seeking, these ticks crawl to the top of vegetation and maneuver their first two legs in anticipation of a passing bloodmeal host. Typically, this behavior is shown in generalist hard tick species, such as *I. scapularis*, *I. pacificus*, *Dermacentor occidentalis* (Say), *Dermacentor andersoni* (Stiles), *A. americanum*, and *Amblyomma maculatum* (Koch) (Eisen and Paddock 2020). One-host ticks, or ticks that spend their complete life cycle on a single host such as *Rhipicephalus annulatus* (Say), and nidicolous ticks do not typically exhibit questing behavior (Furman and Loomis 1984) due to a strong association with single or limited set of hosts. Host specificity and lack of questing behavior minimize the threat of one-host and nidicolous ticks in the transmission of pathogens to humans and other domestic or agricultural hosts. In contrast, questing tick species are often generalists in their host associations, and therefore pose a greater risk of pathogen transmission to susceptible hosts. However, even within the same tick species complex, questing behavior, and therefore human risk, can vary greatly across different geographic regions (Diuk-Wasser et al. 2006, Teel et al. 2010, Hamer et al. 2012, Salkeld et al. 2014, Arsnoe et al. 2019). For example, *I. scapularis* tends to quest more frequently and to higher heights in the northern United States relative to the southern United States (Arsnoe et al. 2019), a behavioral difference that correlates with the high incidence of human Lyme disease in the north relative to the south. Differences in tick questing behavior are likely attributed to variation in climate, host feeding patterns, and tick genetics.

Two of the most commonly used sampling techniques for host-seeking hard ticks are dragging and flagging. These methods are used for the active collection of ticks, and although they remove ticks from the environment, they are not recognized to have an effect on diminishing or controlling tick populations (Daniels et al. 2000, Tälleklint-Eisen and Lane 2000). Both methods exploit the questing behavior by dragging or sweeping a heavy cloth across leaf litter or vegetation to pick up host-seeking ticks (Waladde and Rice 1982, Furman and Loomis 1984, Steullet and Guerin 1992). These two methods are often used in the literature interchangeably but are actually distinct sampling techniques, usually motivated by different objectives (Carroll and Schmidtmann 1992, Dantas-Torres et al. 2013, Rulison et al. 2013, Newman et al. 2019). In drag sampling, the collector moves along an established transects with the drag cloth trailing behind them and in contact with the vegetation, resulting in a known sampling area that can therefore generate estimates of tick density. Accordingly, dragging is the method of choice when one wishes to calculate epidemiologically important metrics including the density of nymphs (DONs) and density of infected nymphs (DINs) (Ostfeld et al. 2006, Gatewood et al. 2009, Diuk-Wasser et al. 2012, Pepin et al. 2012, Eisen and Paddock 2020). These metrics can then be compared between sites and over time (Guerra et al. 2002, Brownstein et al. 2005). In contrast, the flagging technique uses a similar device as the previously described drag cloth, but more closely resembles a flag attached to a pole. The collector uses this adapted flagging device by waving it over vegetation, representing the motion of waving a flag (Dantas-Torres et al. 2013, Rulison et al. 2013). The benefit of flagging is that the collector has more control over the cloth and dowel so the technique can be used in a more targeted manner to collect a certain species or life stage of tick, especially adult ticks, and usually aims to collect a minimum sample size of ticks. Flagging is generally used to determine the presence or lack of detection of ticks (Burgdorfer et al. 1985, Liz et al. 2001) and tick density is not typically evaluated using this method. Further, flagging is also more difficult to standardize between collectors, as sampling effort is not standardized, adding challenges to comparisons of results of different flagging studies (Kugeler and Eisen 2020).

The Centers for Disease Control and Prevention recently issued suggested procedures for *Ixodes* surveillance (Eisen et al. 2019). Building upon the CDC guidelines, we propose a standardized tick dragging protocol with specific attention to sampling area, standardizing the sampling interval distance, and uniform reporting of tick density. The goal of this paper is to provide a standardized tick dragging protocol, which could be useful to new tick researchers or those with an interest in ensuring that relevant data are collected in a standardized way to facilitate comparisons to other studies. We also provide detailed instructions on how to construct a standard tick drag cloth, include a step-by-step protocol for how to set up a linear transect, and provide a template for a field data sheet that can be adapted to suit the needs of individual research projects.

## Protocol

**Fig. 1.**
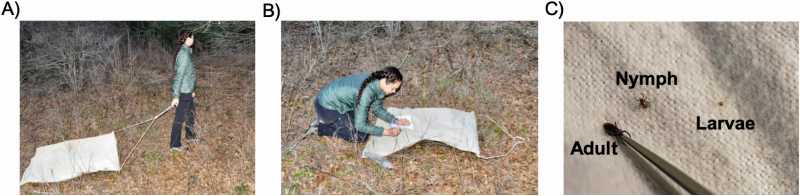
Demonstration of tick dragging in the field. The series of panels show (A) walking to the pace of the ‘wedding march’ (approximately 50 m/min, a 30-min mile, or 1.8 miles/h) with the drag cloth trailing behind the collector, (B) pausing after 15 m to remove any collected ticks, and (C) a size comparison of *Ixodes pacificus* at each life stage to demonstrate the differences in size of the three tick life stages. Photographs by Andrea Swei and Sam McDuff.

Overall, the method of tick dragging involves walking through the designated area to be sampled with a 1-m by 1-m^2^ white cloth dragging behind the collector (Fig. 1A). The size of the cloth allows for determination of the area sampled; the distance of meters dragged is multiplied by the size of the cloth in order to determine the total area dragged, which is needed to determine ticks per unit area. After dragging for 15 m, the collector stops to check the drag cloth and remove any ticks which can be killed and preserved by placing into 70–95% ethanol, or kept alive by placing into small chambers that allow for humidity and air exchange. To drag sample a predetermined area of at least 750 m^2^, a grid or linear transects can be established. Drag sampling along the same grid or transect can be done repeatedly for longitudinal studies to define host-seeking phenology or compare year-to-year density, for example.

### Building a 1-m^2^ Drag Cloth

#### Materials

- Sewing machine, or a sewing needle, size 16 US or 100 UK
- Thick thread (2-ply bonded nylon Tex 60)
- Heavy cotton flannel fabric that is white or another light color to facilitate visibility of ticks. The dimensions should be a minimum of 1.25 yards by 1.25 yards (114 cm by 114 cm). Wash the material with fragrance-free soap before cutting and sewing, to allow for material shrinkage.
- Sewing pins
- Wooden dowel 1″ diameter (2.4 cm). In the United States, dowels are typically sold in 45″ lengths (1.143 m).
- Nylon rope with a thickness of 0.5″ (1.27 cm) for attachment to the dowel. The length at which the rope is cut depends on the height of the individual, but generally we recommend about 3 m of rope per drag cloth, in order to fasten the rope to the dowel and provide length for the entire cloth to comfortably drag on the ground, about a half a meter behind the tick collector.
- An electric drill with a 9/16″ (14.5 mm) drill bit to accommodate for the width of the rope used. The hole for the rope can also be screwed by hand.
- Metal fishing weights (‘sinkers’) or other small weights to add approximately 8–12 ounces of total weight to the bottom of the drag cloth. Another option alternative to the individual weights is to insert a 90-cm metal chain. The chain method over the free weights may prevent the weights from moving inside the hem.

#### Instructions

**Fig. 2.**
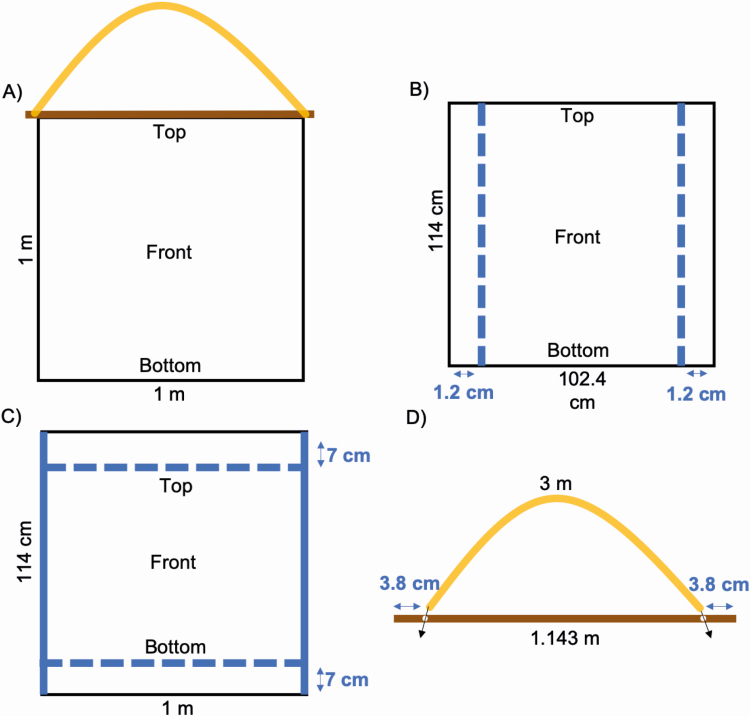
Diagram illustrating how to construct a drag cloth including (A) the final drag cloth design, (B) hemming the sides by 1.2 cm, (C) building the enlarged hems for the dowel slot and the weights pocket, and (D) attaching the rope to the dowel. The front is the ‘clean’ side with no hem fringe and comes in contact with the leaf litter or vegetation (Fig. 1A). The back is the side that will face upwards when being dragged. By maintaining the distinction of each side of the drag cloth, the hems will remain intact longer and will reduce the risk of overlooking larvae lost inside the fringes of the hem.

1) Orient the fabric with a top, bottom, front, and back (Fig. 2). Measure and cut the cloth to a width of 102.4 cm and length of 114 cm.
2) Create the hem by folding in 1.2 cm from the sides and pin down with sewing pins (Fig. 2B). The fringe of the hem is the back side. Once sewing is complete, remove the pins.
3) To create the hemmed top loop and the bottom pocket, for both dowel and weights, measure and fold in 7 cm from the top and bottom edge of the drag cloth and pin. The fringe of the top and bottom hem should also be on the back side. The hemmed top loop and bottom pocket will both be 7 cm, resulting in a finished area of 1 m by 1 m.
4) Insert the chosen material of weight at the bottom pocket and sew the edges closed. For ease of laundering, the bottom pocket can be fastened with buttons or Velcro, rather than sewn shut, to allow for the weights to be removed before cleaning. To better secure the free individual weights, a few vertical lines can be sewn in the hem to prevent the free weights from moving around.
5) Insert the dowel in the top hem loop. The ends of the dowel should be slightly longer than the drag cloth on both sides to attach the rope. By leaving the top hem loop open, this allows for easy removal of the cloth from the dowel before washing.
6) Drill a hole through the dowel 3.8 cm from each end. The hole should be big enough to accommodate the rope.
7) Cut the rope to a length of 3 m and fasten it through the holes of the dowel (Fig. 2A). This rope length can be adjusted according to the height of the collector to maintain a 45° angle between the drag cloth and the surface being dragged by tying a knot to reduce the length. Tie the rope in a sturdy knot that can be untied in order to easily remove the cloth for cleaning (Fig. 2D).

#### Considerations for Drag Cloth Material

Corduroy is also commonly used in drag cloths. The fabric wales (ridges) increase surface area such that the sampling cloth size may be greater than 1 m^2^ which may be a consideration in density estimates. Additionally, depending on wale size, larvae may be overlooked as they are quite small and can hide in the crevices of the corduroy (Fig. 1C). Fabrics such as muslin have had better success in collecting *A. americanum* (Newman et al. 2019), but this material may need to be replaced more often due to its relative thinness. Denim may also be considered, but make sure that it is light enough in order to see the ticks (Eisen et al. 2019). In congruency with the CDC, we recommend using a thick white flannel material, as it is easy to wash, durable, less expensive, and easier to find at a local fabric store. When soiled, the drag cloth can be laundered (with dowel and weights removed) with non-fragrance laundry soap in a regular washing machine system.

### Establishing a Linear Transect

#### Materials

- Compass
- Fiberglass open reel field tape measure
- Stake flags

#### Instructions

**Fig. 3.**
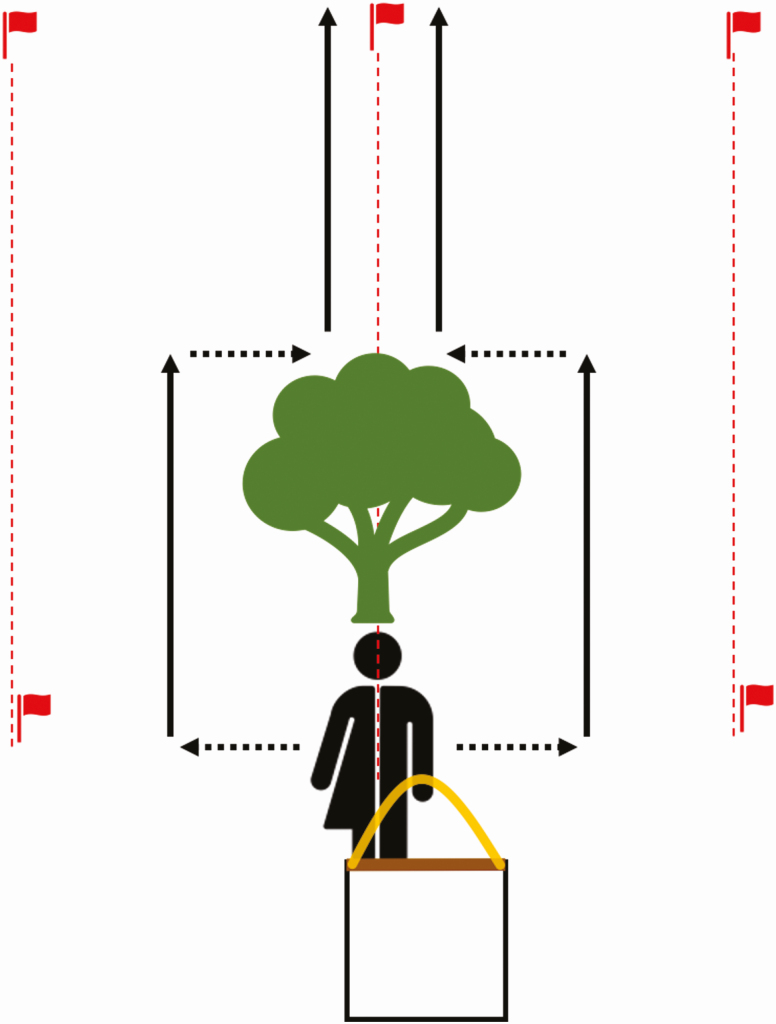
Illustration showing how to maneuver around a large obstacle for drag sampling. Three parallel linear transects are depicted (red dashed lines), with stake flags designating 15 m intervals. The middle transect has a tree obstructing the linear path. When maneuvering around the obstruction, dragging should continue only when moving parallel to the original transect (solid black lines), but not when maneuvering to the left or right around the obstruction (dashed black lines).

1) Establish linear transects that total a minimum of 750 m in length using a field tape measure and a compass to ensure linearity.
2) While the field tape measure is uncoiled, demarcate every 15 m along the transect from start to finish with stake flags. The transect can be a single transect line or several parallel transect lines (e.g., 10 parallel transects of 75 m; Supp Table 1 [online only]). Make sure to space out the parallel transects with at least 10 m in between, in case there is an obstruction (Fig. 3).

#### Considerations of Habitat

Habitat type can have a large effect on the densities and species compositions of ticks so this is an important detail to report (Guerra et al. 2002; Eisen et al. 2003, 2005; Civitello et al. 2008; Williams and Ward 2010; Nakano 2014; MacDonald et al. 2017; Lerman and D’Amico 2019). Dragging along trails makes accessibility easier and samples habitat where hikers are most likely to encounter ticks and may be suitable for some studies. Further, as many large wildlife hosts use trails, ticks may be more common along trails relative to the interior of the forest (Muhly et al. 2011, Erb et al. 2012, Kays et al. 2017). However, unless the surveillance or research objective necessitates dragging specifically along trails, we recommend establishing linear dragging transects that may start along trails but include interior habitats (Maupin et al. 1991, Eisen et al. 2019, Lerman and D’Amico 2019). As an example, Diuk-Wasser et al. (2006), used GIS and maps of a parks trails to establish a set of random points on trails to initiate tick dragging, and transects continued into the interior habitats of the forest. Then to determine the direction of the parallel transects from the starting point, a number was randomly selected to determine the angle relative to the hiking trail, after which a linear path was followed. Furthermore, consider dragging within preferred habitats of the targeted species. For example *Ixodes* species are more predominantly found within leaf litter under a high percentage of canopy cover (Lane and Stubbs 1990, Schulze et al. 1995, Guerra et al. 2002), whereas *Amblyomma* and *Dermacentor* species are more predominantly found in open grasslands (Schulze et al. 2002, MacDonald 2018).

#### Considerations of Sampling Interval

Sampling interval, or the sampling distance at which drag cloths are checked, vary across studies ranging from 5 to 30 m among studies which report it (Jones et al. 1998, Perret et al. 2000, Randolph et al. 2002). Studies have shown that past about 15 m of sampling distance, ticks begin to detach from the drag cloth (Li and Dunley 1998, Eisen et al. 2019). Therefore, it is important that surveillance efforts adopt a standard distance of 15-m intervals to check drag cloths so that density estimates are comparable (Borgmann-Winter and Allen 2019). Placing stake flags along the transect are useful reminders of when to stop and check the drag cloth for ticks.

#### Considerations to Size of Area Sampled

The area sampled for estimating tick densities is an important consideration, especially when data from multiple field efforts are compared to assess tick-borne disease risk. Studies report a wide range of total sampling area, from 400 m^2^ up to 74,000 m^2^ per site (Maupin et al. 1991, Allan et al. 2003). Although ticks are known to have patchy distributions across the landscape (Horobik et al. 2007, Perez et al. 2016, VanAcker et al. 2019), we are not aware of studies that specifically assess the scale at which differences in area sampled may affect tick density estimates. Although CDC guidelines (Eisen et al. 2019) and our protocol recommend sampling 750 m^2^, smaller sites may not allow for this area of drag sampling (e.g., small neighborhood parks). A key aspect is that researchers should report the total area sampled, but more importantly report tick density as average number of ticks per 100 m^2^. This will allow for standardization of tick density across studies, despite variable sampling area. Future studies should investigate the effect of variation in drag sample area on tick density estimates.

### Tick Dragging

#### Materials

- Fine tipped forceps
- Pale 7.4-cm masking tape or a lint roller with clear plastic, sealable bags (for abundant larvae collection)
- Collection tubes, with 70–95% ethanol for lethal collection if desired. Consider the molecular objectives and storage timeframe of the samples for which ethanol concentration to use, but refrain from denatured ethanol as it can interfere with PCR (Hubbard et al. 1995, Mtambo et al. 2006, Ammazzalorso et al. 2015, Eisen et al. 2019).
- A datasheet (Supp Table 1 [online only]) or field notebook
- Drag cloth (see above)

#### Instructions

1) Avoid wearing any insecticides while collecting ticks. Furthermore, avoid drag sampling when the vegetation is wet or too dry, as this will impair tick attachment to cloth and tick activity in general (Russell and Jain-Sheehan 2015, Eisen et al. 2019). It is notable that some ticks peak in activity in the evening or at night, but this is not necessarily a practical time to sample for safety and tick visibility reasons (Durden et al. 1996).
2) Starting at the beginning of the first transect, the drag cloth should be laid flat on the ground.
3) The transect line is sampled at the pace of the typical ‘wedding march’ (approximately 50 m/min; a 30-min mile; or 1.82 miles/h) in a single direction and as straight as possible (Fig. 1A). Please note that the time spent checking the cloth for ticks is not factored into the pace specified above. Adhering to this pace over a known distance is easier to standardize rather than sampling for an allotted amount of time (Russell and Jain-Sheehan 2015). The drag cloth should remain flat on the ground throughout the sampling.
4) If there is an obstruction, such as a large log or a tree within a transect, sampling should not go over or around the obstruction while drag sampling. Instead, the drag cloth should be picked up, moved to the left or right to clear the obstruction, and then sampling can continue in a line parallel to the original transect line (Fig. 3). If possible, it is better to be consistent with which direction a collector shifts to of the obstruction to reduce bias. Once the obstruction is passed, dragging on the original transect line can resume. By going around smaller obstacles such as logs, sampling is more standardized between sampling sessions and loss of ticks from friction is minimized.
5) Once 15 m have been covered, dragging should be paused and all attached ticks to the 1-m^2^ drag cloth area should be removed (Fig. 2B). Care should be taken to carefully check the front, back, and fringes of the cloth, including the hem in a systematic routine. Ticks found on the rope or the collector may be saved, but keep in a separate collection tube to not be confused as a part of the standardized dataset (Fig. 4). The best way to see some of the larvae is to look for the shine of the scutum in the sunlight.
6) Ticks should be removed with fine tipped forceps and placed into a sample vial of 70–95% ethanol (Hubbard et al. 1995, Mtambo et al. 2006, Ammazzalorso et al. 2015, Eisen et al. 2019). To collect a large number of larvae at a time, tape can be used to pick up the ticks from the cloth and transfer them to a plastic bag or sealable container (Daniels et al. 2000). If the collection of live ticks is needed, individual larvae can be gently removed from the tape with forceps and transferred to a sample vial and then stored under appropriate humidity conditions for the species in question (Winston and Bates 1960, Roggendorf et al. 2015, Levin and Schumacher 2016).
7) A field data sheet should be used to continuously record tick abundances per transect (Supp Table S1 [online only]). This will be useful for verifying abundances when collections are assessed in the laboratory. We also recommended using a new tube for each transect if there are multiple transects.
8) After all ticks have been removed, dragging can continue over the next 15-m sampling interval. Repeat as above until the entire transect has been sampled. If there are multiple transects, the drag cloth should be picked up and walked to the next transect where dragging can resume.
9) It is also important to practice proper tick safety when collecting ticks such as routine tick checks throughout the time in the field, showering as soon as possible post collection, and drying the worn clothes in a dryer on a high heat cycle for at least 10 min (Nelson et al. 2016, Eisen et al. 2019).

#### Considerations of Tick Phenology

Pilot studies to determine the peak seasonal period of tick questing in the region of interest may be useful if the goal is to then concentrate sampling efforts during peak activity periods. For instance, *I. scapularis* in different regions of the United States, have different phenologies. In the Northeast, *I. scapularis* nymphs peak in their emergence is a month before the larvae, while in the Midwest, the two life stages have a synchronous peak emergence in the summer (Diuk-Wasser et al. 2006, Hamer et al. 2012). Similarly, A. maculatum populations in Oklahoma and Kansas are active approximately five months earlier than populations of southeast Texas (Teel et al. 2010).

## Conclusion

Tick-borne diseases are an emerging global threat and are still not fully understood (Jones et al. 2008, Rosenberg et al. 2018, Swei et al. 2020). Standardization in tick surveillance methodology and reporting would facilitate aggregation of data across studies and allow for geographic comparisons of tick density and entomological/acarological risk assessments, greatly benefiting the field.

Tick dragging often results in a dataset comprised of multiple life stages and species of ticks (Furman and Loomis 1984, MacDonald 2018, MacDonald et al. 2020). From dragging data collected in a standardized manner, one can calculate the abundances and densities of each life stage at that given time. Furthermore, pathogen testing of the collected ticks from a known area sampled allows for the calculation of informational metrics of disease risk, such as DINs and nymphal infection prevalence (NIP) (Eisen et al. 2019, Kugeler and Eisen 2020). Metrics such as DIN and NIP collected at the same sites annually can provide longitudinal data on tick population fluctuations and entomological disease risk. These data can then be assessed with multivariate modeling to develop predictive models of long-term disease risk (LoGiudice et al. 2003, Ostfeld et al. 2006, Diuk-Wasser et al. 2012).

Although many studies report tick abundance standardized as tick density per 100 m^2^ or ticks per 1,000 m^2^, several studies report abundance but not density (Maupin et al. 1991, Kurtenbach et al. 1998, Stafford et al. 1998, Allan et al. 2003). We encourage researchers to be transparent in reporting the area sampled, abundance of ticks collected for each species of each life stage, and the density of each tick species and life stage as the average number of ticks per 100 m^2^, so that data can be shared and compared across studies.

Throughout the literature, tick surveillance studies employ numerous methods of tick collection and data reporting. The lack of a standardized best practice limits comparative studies and hampers the study of tick-borne diseases, a growing national health concern (Guerra et al. 2002, Little et al. 2019, Swei et al. 2020). By providing this protocol and considerations for standardizing drag sampling, with the key considerations of 1) tracking area sampled, 2) sampling at intervals of 15 m, and 3) transparency in data reporting, our goal is to optimize tick surveillance to improve our understanding of spatial and temporal trends in tick population dynamics and for controlling the emergence of tick-borne diseases (Eisen et al. 2019, Eisen and Paddock 2020, Kugeler and Eisen 2020).

## Supplementary Material

ieaa073_suppl_Supplementary_Table_S1
