# Supplementary material for: A Beginner’s Guide to Collecting Questing Hard Ticks (Acari: Ixodidae): A Standardized Tick Dragging Protocol
Source: J Insect Sci. 2020 Nov 2;20(6):11. doi: 10.1093/jisesa/ieaa073 (PMC7604844; doi:10.1093/jisesa/ieaa073)
Supplement: ieaa073_suppl_Supplementary_Table_S1 [file ieaa073_suppl_supplementary_table_s1.docx]

**Supplemental Table S1** Sample field datasheet to track tick collections over a 750m^2^ drag area. Data sheet can be modified by individual users to meet research needs. It is recommended to record ticks at 15 m intervals, using either a tally mark system or just writing the numbers and adding them up for each life stage at the end of the transect. Tick species and life stage should be verified under a dissecting microscope in the lab.

| **Date** | | | | | **Sampling Start time** | | | | | | | | | | |  |  |  |  |  |
| --- | --- | --- | --- | --- | --- | --- | --- | --- | --- | --- | --- | --- | --- | --- | --- | --- | --- | --- | --- | --- |
| **Collector** | | | | | **Sampling end time** | | | | | | | | | | |  |  |  |  |  |
| **Site** | | | | | **Weather** | | | | | | | | | | |  |  |  |  |  |
|  | **1 to 15 meters** | | | **15 to 30 meters** | | | **30 to 45 meters** | | | **45 to 60 meters** | | | | **60 to 75 meters** | | | | |  |  |
| **Transects** | **L** | **N** | **A** | **L** | **N** | **A** | **L** | **N** | **A** | **L** | **N** | **A** | **L** | | **N** | | **A** | **Total** | |  |
| **A** |  |  |  |  |  |  |  |  |  |  |  |  |  | |  | |  |  | |  |
| **B** |  |  |  |  |  |  |  |  |  |  |  |  |  | |  | |  |  | |  |
| **C** |  |  |  |  |  |  |  |  |  |  |  |  |  | |  | |  |  | |  |
| **D** |  |  |  |  |  |  |  |  |  |  |  |  |  | |  | |  |  | |  |
| **E** |  |  |  |  |  |  |  |  |  |  |  |  |  | |  | |  |  | |  |
| **F** |  |  |  |  |  |  |  |  |  |  |  |  |  | |  | |  |  | |  |
| **G** |  |  |  |  |  |  |  |  |  |  |  |  |  | |  | |  |  | |  |
| **H** |  |  |  |  |  |  |  |  |  |  |  |  |  | |  | |  |  | |  |
| **I** |  |  |  |  |  |  |  |  |  |  |  |  |  | |  | |  |  | |  |
| **J** |  |  |  |  |  |  |  |  |  |  |  |  |  | |  | |  |  | |  |
| **Total** |  |  |  |  |  |  |  |  |  |  |  |  |  | |  | |  |  | |  |
| **Were any ticks removed from rope or collector during the sampling session? If so, how many? What is the collection tube labeled?** | | | | | | | | | | | | | | | | | | | | |
